# *Brucella* Periprosthetic Joint Infection Involving Bilateral Knees with Negative Synovial Fluid Alpha-Defensin

**DOI:** 10.1155/2019/9423946

**Published:** 2019-07-14

**Authors:** Abdullah Balkhair, Sultan Al Maskari, Shadin Ibrahim, Ibrahim Al Busaidi, Mohammed Al Amin, Hashim Ba Taher

**Affiliations:** ^1^Department of Medicine, Infectious Diseases Unit, Sultan Qaboos University Hospital, P.O. Box. 35, Al koudh. 123, Muscat, Oman; ^2^Department of Surgery, Orthopaedics Unit, Sultan Qaboos University Hospital, P.O. Box. 35, Al koudh. 123, Muscat, Oman

## Abstract

Periprosthetic joint infection (PJI) due to *Brucella* is uncommon despite relatively high endemicity of human brucellosis and its osteoarticular predilection. We report a case of a 57-year-old woman with bacteraemic brucellosis complicated by *Brucella* periprosthetic infection of both knee joints occurring a decade after bilateral knee arthroplasty and associated with a negative synovial fluid alpha-defensin test. The patient was successfully treated with anti-*Brucella* therapy alone and without surgical revision, resulting in clinical and microbiological cure. We propose that *Brucella* should be considered as a possible cause of prosthetic joint infection in the appropriate clinical and epidemiological settings. A negative synovial fluid alpha-defensin (Synovasure AD test) should not be used as a rule-out test for *Brucella* PJI. *Brucella* PJI without radiological loosening may be treated conservatively and solely with antimicrobial therapy.

## 1. Introduction

Brucellosis is a zoonotic infection with approximately 500,000 cases reported annually in disease-endemic regions [1]. Brucellosis is caused by any of the four pathogenic species to humans, namely, *Brucella abortus*, *B. melitensis*, *B. suis*, and *B. canis*. Although brucellosis is believed to be a significant livestock infection in Oman, it has not been studied extensively [2, 3]. Ingestion of unpasteurized milk or milk products is believed to be the commonest route of transmission in Oman [4]. A retrospective analysis of human brucellosis in Oman between 1995 and 2012 identified 2737 human cases of brucellosis with 96.7% of these in the southern part of Oman (Dhofar) [4]. Brucellosis is characterized by its myriad presentations and multisystemic nature with osteoarticular involvement being one of the most frequent manifestations of this disease [5].

Total knee arthroplasty is one of the most common procedures in orthopaedic surgery including in areas where brucellosis is endemic [6] with periprosthetic joint infection (PJI), one of the most serious complications of prosthetic joint implantation [7]. Periprosthetic joint infections may account for up to 25% of revision total knee arthroplasties [8].

Periprosthetic joint infection due to *Brucella* is infrequent. In a recent review of published literature of *Brucella* PJIs, thirty cases were identified of which nineteen cases were prosthetic knee infections including four cases with *Brucella* infection of bilateral knee prostheses [9]. The median time from implantation of the prosthesis to the diagnosis of PJI was two years in this same cohort [9]. The pathogenesis of *Brucella* PJI is believed to be due to hematogenous seeding of the arthroplasty components complicating bacteraemic brucellosis [10].

Diagnosis of periprosthetic joint infection—albeit *Brucella* PJI—can be challenging. However, several publications exist to guide diagnosis of PJI in general, and these include guidelines by the Musculoskeletal Infection Society [11], the International Consensus Group on Periprosthetic Joint Infection [12], the 2018 Definition of Periprosthetic Hip and Knee Infection [13] where new diagnostic tests were incorporated, and the World Association against Infection in Orthopaedics and Trauma (WAIOT) proposed new definition for PJI based on diagnostic tests/procedures, clinical presentation, and intraoperative/postoperative confirmation [14]. WAIOT recognizes postoperative histological and microbiological analysis as key procedures to confirm or to exclude the diagnosis of PJI. In addition, WAIOT suggests that novel diagnostic tests may be implemented in the definition of PJI if they meet the required sensitivity and/or specificity thresholds [14]. One of these novel diagnostic tests for PJI is synovial fluid alpha-defensin [15]. Alpha-defensin is an antimicrobial peptide that is secreted by neutrophils in response to presence of pathogens [16]. Intraoperative detection of alpha-defensin in synovial fluid is facilitated by use of a lateral flow device (Synovasure AD test) with results available in just ten minutes. This test is reported to have an overall sensitivity of 92.1% and a specificity of 100% [17]. Accordingly, it was proposed that a lateral flow device (Synovasure AD test) should not be used for screening, but rather as a confirmatory test for PJI [18]. The role of biomarkers including synovial fluid alpha-defensin in the diagnosis of *Brucella* PJI is unknown and to our knowledge has not been studied. We believe that the fastidious nature of *Brucella* often resulting in negative culture of synovial fluid in *Brucella* PJI argues favourably to examine whether biomarkers can assist in this challenging diagnosis.

There is currently no consensus on how best *Brucella* PJI should be treated with regard to surgical intervention or duration of antimicrobial therapy. It is suggested based on a review of 24 cases with *Brucella*-related PJI that in the absence of radiological evidence of joint loosening, antimicrobial therapy alone may be appropriate [19].

## 2. Case Presentation

A 57-year-old woman underwent bilateral total knee arthroplasty for treatment of severe degenerative joint disease 10 years prior to her current presentation. She presented to our hospital with progressively worsening pain and swelling of both prosthetic knee joints for six weeks. She also reported a history of indolent fever, night sweats, and malaise started one week prior to joint symptoms. She was recently diagnosed with deep vein thrombosis of the left femoral vein for which she is on rivaroxaban. She reported consumption of possibly unpasteurized milk four weeks prior to her illness.

**Figure 1:**
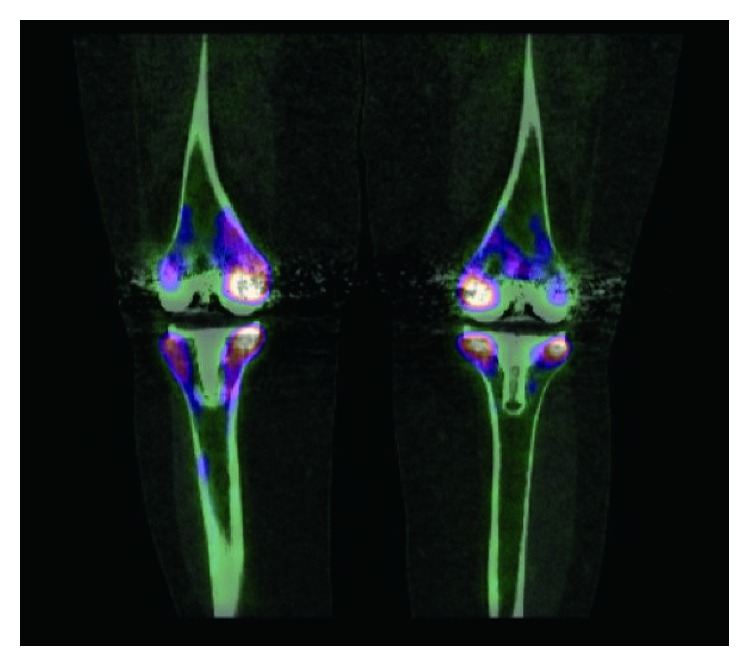
SPECT/CT image of bilateral prosthetic knees demonstrating increased radiotracer uptake around both knee joint prostheses compatible with periprosthetic joint infection.

Examination revealed a lethargic, febrile woman with bilateral swollen, warm, and tender knee joints with moderate effusions. Rest of examination was normal. Initial investigations showed a hypochromic microcytic anaemia with a haemoglobin of 9.4 g/dl, a normal total white cell count (5600 cells/mm^3^), and elevated inflammatory biomarkers with an erythrocyte sedimentation rate (ESR) of 109 mm/h and C-reactive protein (CRP) of 101 mg/dL. Single-photon emission computed tomography/computed tomography (SPECT/CT) of both knees is shown in Figure 1.

Diagnostic aspiration of both knee joints was performed. Analysis of the synovial fluid revealed 8200 white cells/*μ*L with predominance of lymphomononuclear cells. Lateral flow alpha-defensin (Synovasure AD test) was performed intraoperatively on fluids from both knee joints and was negative twice. Meanwhile, a blood culture that was sent on admission grew *Brucella melitensis* after 6 days of incubation. Subsequently, culture of the synovial fluid (from both joints) grew *Brucella melitensis* on day 8 of incubation. A diagnosis of bacteraemic brucellosis with brucellar bilateral prosthetic knee joint infection was established, targeted antimicrobial therapy was commenced with combination of intravenous gentamicin 5 mg/kg once daily (for one week) in addition to oral doxycycline 100 mg twice daily and oral rifampicin 600 mg once daily (both given for 24 weeks), and a rescue revision arthroplasty was planned contingent on the outcome of antimicrobial therapy. In view of the chronicity of symptoms and associated bacteraemia, the patient underwent a transthoracic echo which showed no vegetations. Subsequent follow-up sterility blood cultures were negative. By end of first week of antimicrobial therapy, the patient was able to walk unassisted, and at 3 months, she had complete resolution of symptoms with normalization of inflammatory biomarkers; hence, a revision surgery was not performed. The patient remained symptom-free three years later with no evidence of disease recurrence or relapse.

## 3. Discussion

Periprosthetic joint infection (PJI) due to *Brucella* is uncommon and limited to few case reports despite relatively high endemicity of human brucellosis and its osteoarticular predilection. To our knowledge, our case—with bilateral knee periprosthetic *Brucella* infection—is the fifth case in the published literature [9] and the first report from Oman. Furthermore, our patient had bacteraemic bilateral knee *Brucella* PJI occurring 10 years after bilateral knee arthroplasty denoting one of the extremely late *Brucella* PJIs ever reported [9]. Unlike our case, diagnosis of *Brucella* PJI can be challenging in the absence of systemic symptoms and epidemiological clues partly due to the fastidious nature of the organism and the nonspecific synovial fluid findings. Synovial fluid alpha-defensin testing is an innovative diagnostic test for PJI [15]. However, its role in the diagnosis of *Brucella* PJI is unknown and has not been studied. In our patient, we used the lateral flow device (Synovasure AD test) intraoperatively for detection of alpha-defensin in synovial fluid from both knee joints. Both samples tested falsely negative. Arguably, this is the first published report on the use of Synovasure AD test for detection of alpha-defensin in synovial fluid from *Brucella* PJI. We propose to examine the role of alpha-defensin and other biomarkers in this infrequent but challenging diagnosis. According to WAIOT, novel diagnostic tests may be implemented in the definition of PJI if they meet the required sensitivity and/or specificity thresholds [14]. This is currently unknown for synovial fluid alpha-defensin in *Brucella* PJI.

Treatment of *Brucella* PJI is equally challenging. There is no consensus on how best *Brucella* PJI should be treated. Published case reports of *Brucella*-related PJI suggest that in the absence of radiological evidence of joint loosening, standard anti-*Brucella* antimicrobial therapy alone may be appropriate [19]. Our patient did not have evidence of joint loosening; hence, she was exclusively treated with anti-*Brucella* antimicrobial therapy, resulting in sustained clinical and microbiological cure without the need for revision surgery further supporting current dogma.

## 4. Conclusion

We reported a case of bacteraemic brucellosis complicated by late *Brucella* periprosthetic infection of both knee joints with negative synovial fluid alpha-defensin and successfully managed with antimicrobial therapy alone. We propose that *Brucella* should be considered as a possible cause of prosthetic joint infection in the appropriate clinical and epidemiological settings and a negative synovial fluid alpha-defensin (Synovasure AD test) should not be used as a rule-out test for *Brucella* PJI.
